# Durable regression of Medulloblastoma after regional and intravenous delivery of anti-HER2 chimeric antigen receptor T cells

**DOI:** 10.1186/s40425-018-0340-z

**Published:** 2018-04-30

**Authors:** Anandani Nellan, Christopher Rota, Robbie Majzner, Cynthia M. Lester-McCully, Andrea M. Griesinger, Jean M. Mulcahy Levy, Nicholas K. Foreman, Katherine E. Warren, Daniel W. Lee

**Affiliations:** 1 Department of Pediatrics, University of Colorado Anschutz Medical Campus, Aurora, CO USA; 2 Morgan Adams Foundation Pediatric Brain Tumor Research Program, Children’s Hospital Colorado, Aurora, CO USA; 3 Division of Medical Sciences, Harvard Medical School, Harvard University, Boston, MA USA; 4 Division of Pediatric Hematology and Oncology, Department of Pediatrics, Stanford University, Stanford, CA USA; 5 Pediatric Oncology Branch, Center for Cancer Research, National Cancer Institute, National Institutes of Health, Bethesda, MD USA; 6 Division of Pediatric Hematology and Oncology, Department of Pediatrics, University of Virginia, PO Box 800386, Charlottesville, VA 22908 USA

**Keywords:** Medulloblastoma, Chimeric antigen receptor T cell, HER2, Nonhuman primate

## Abstract

**Background:**

Standard-of-care therapies for treating pediatric medulloblastoma have long-term side effects, even in children who are cured. One emerging modality of cancer therapy that could be equally effective without such side effects would be chimeric antigen receptor (CAR) T cells. Knowing that human epidermal growth factor receptor 2 (HER2) is overexpressed in many medulloblastomas and has been used as a CAR T target before, we sought to evaluate the efficacy of more sophisticated anti-HER2 CAR T cells, as well as the feasibility and efficacy of different routes of delivering these cells, for the treatment of pediatric medulloblastoma.

**Methods:**

Daoy, D283 and D425 medulloblastoma cell lines were characterized by flow cytometry to evaluate HER2 expression. Anti-tumor efficacy of HER2-BBz-CAR T cells in vitro was performed using cytokine release and immune cytotoxicity assays compared to control CD19 CAR T cells. In vivo, Daoy and D283 tumor cells were orthotopically implanted in the posterior fossa of NOD.Cg-*Prkdc*^*scid*^ *Il2rg*^*tm1Wjl*^/SzJ (NSG) mice and treated with regional or intravenous HER2-BBz-CAR T cells or control CD19 CAR T cells. Non-human primates (NHPs) bearing ventricular and lumbar reservoirs were treated with target autologous cells bearing extracellular HER2 followed by autologous HER2-CAR T cells intraventricularly. Cerebrospinal fluid and blood were collected serially to measure the persistence of delivered cells and cytokines.

**Results:**

HER2-BBz-CAR T cells effectively clear medulloblastoma orthotopically implanted in the posterior fossa of NSG mice via both regional and intravenous delivery in xenograft models. Intravenous delivery requires a log higher dose compared to regional delivery. NHPs tolerated intraventricular delivery of autologous cells bearing extracellular HER2 followed by HER2-BBz-CAR T cells without experiencing any systemic toxicity.

**Conclusions:**

HER2-BBz-CAR T cells show excellent pre-clinical efficacy in vitro and in mouse medulloblastoma models, and their intraventricular delivery is feasible and safe in NHPs. A clinical trial of HER2-BBz-CAR T cells directly delivered into cerebrospinal fluid should be designed for patients with relapsed medulloblastoma.

**Electronic supplementary material:**

The online version of this article (10.1186/s40425-018-0340-z) contains supplementary material, which is available to authorized users.

## Background

Medulloblastoma is the most common type of malignant pediatric brain tumor, comprising approximately 20% of all new diagnoses in children between 0 and 19 years old, but can occur in people of all ages [1]. Current standard of care consists of gross total resection and intensive chemotherapy, with or without radiation [1]. While overall survival with this treatment regimen approaches 75% at 5 years, it carries a high burden of morbidity that includes neurocognitive loss, endocrine abnormalities, hearing loss, growth problems and mortality from secondary malignancies [2]. Recurrent disease is usually fatal because of ineffective salvage therapies [2], and studies exploring alternatives to traditional chemotherapy, such as targeted inhibitors, have not been successful [3]. The development of new modalities of cancer therapy for medulloblastoma is therefore essential to the improvement of patient outcomes.

Receptor tyrosine-protein kinase ERBB2 (HER2) is a well-known immunotherapy target that is overexpressed in multiple adult and pediatric cancers [4–7], including approximately 40% of medulloblastoma. HER2 surface expression on medulloblastoma is associated with significantly worse overall and progression free survival [8, 9]. Additionally, ERBB2 protein is not detected in normal brain [10], which has made it an attractive target for therapy in these tumors. In the treatment of breast cancer, anti-HER2 monoclonal antibodies interfere with HER2 signaling and cause cell death in cancer cells. However, such antibodies have been found to be ineffective against medulloblastomas, partly due to the fact that HER2 surface expression in medulloblastoma is lower than in breast cancer and not associated with HER2 gene amplification [11]. Thus, alternative modalities have been explored to exploit HER2 overexpression, among them chimeric antigen receptor (CAR) bearing T cells.

CAR T cell therapy is a promising approach to target HER2 expressing tumors, as it eliminates cancer cells through direct T cell cytotoxicity rather than relying on antibody-dependent cell-mediated cytotoxicity. T cells bearing CARs combine the specificity of an antibody with the cytolytic ability of a T cell and can effectively kill tumor cells bearing a specific antigen [12]. CD19-directed CAR T cells have produced dramatic responses in patients with relapsed and refractory B cell malignancies [13, 14]. Adoptive transfer of natural killer cells [15] and CAR T cells [11] have both shown promise in mouse models of medulloblastoma. In previous pre-clinical studies, first generation HER2-CAR T cells containing CD3 zeta signaling without a co-stimulatory signaling domain caused only transient tumor regression in an orthotopic xenograft mouse model of medulloblastoma [11]. However, newer generations of CAR T cells incorporating co-stimulatory domains have increased proliferation and persistence in other tumor models, resulting in improved cytotoxic potential [12]. HER2-CARs are currently being used in clinical trials for the treatment of glioblastoma and sarcomas and have proven to be safe and well-tolerated [16, 17].

Here, we show that second generation HER2-CAR T cells containing the CD3 zeta and 4-1BB signaling motifs have robust in-vitro antitumor activity against medulloblastoma cell lines, and eradicate tumors in multiple orthotopic xenograft mouse models of medulloblastoma. We demonstrate that very low doses of HER2-CAR T cells are capable of inducing full and durable regression of established CNS tumors when administered regionally. We also show that these CAR T cells persist at low levels in the brain and peripheral blood of treated mice for an extended period after tumor clearance. Lastly, we show that intraventricular delivery of HER2-CAR T cells in non-human primates (NHPs) is feasible without systemic toxicity and may be an important method of delivery for some patients. This study provides clear evidence of the efficacy of newer generation HER2-CAR T cell therapy against medulloblastoma. Considering the lack of effective salvage regimens for patients with relapsed medulloblastoma, these findings should be rapidly translated into a clinical trial.

## Methods

### Blood donors and cell lines

The medulloblastoma cell lines Daoy and D283 were obtained from American Type Culture Collection (ATCC) and D425 originated in the laboratory of Dr. Darell Bigner (Duke University Medical Center). The ependymoma 928 cell line originated in the laboratory of Dr. Nicholas K. Foreman and was used as a positive control for HER2 surface expression. All cell lines were grown in EMEM (ATCC) with 10% fetal calf serum (FCS) and 1% penicillin-streptomycin-glutamine (PSG) (Life Technologies). All cell lines were verified with short tandem repeat analysis and confirmed mycoplasma free. Cell lines were transduced with a lentivirus containing green fluorescent protein (GFP) and luciferase coding sequences that were co-expressed under the human elongation factor-1 promoter. Cells were flow sorted for GFP expression and then single cell cloned. Human peripheral blood mononuclear cells (PBMCs) were obtained from normal donors at the National Institutes of Health Blood Bank under Institutional Review Board approved protocols. T cells were grown in AIM-V (Life Technologies) with 5% FCS and 1% PSG. All cells were cultured at 37°C and 5% CO2.

### Construction of chimeric antigen receptors genes

**Fig. 1:**
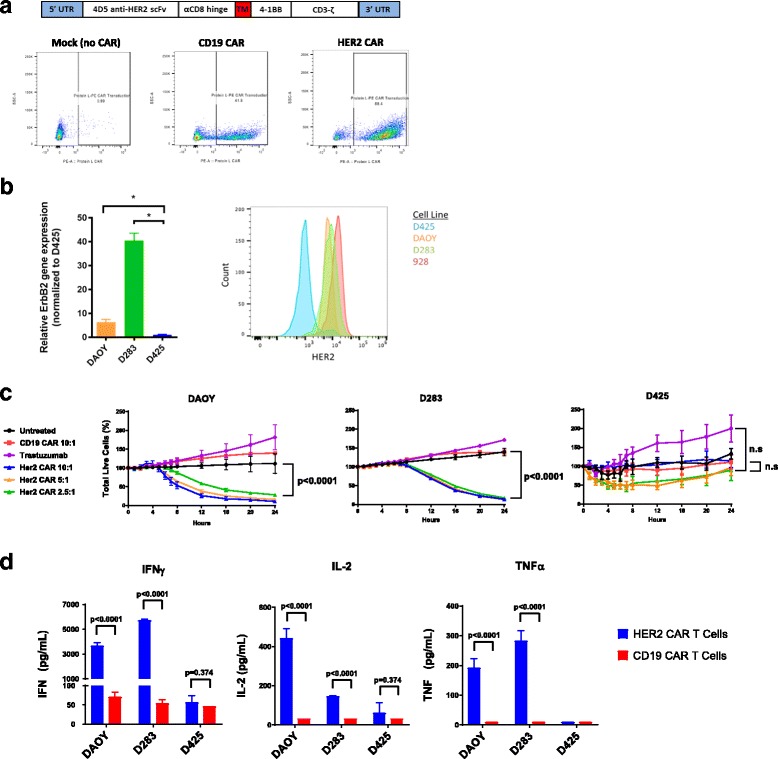
Construction and in vitro evaluation of HER2 CAR T cells. **a** To create anti-HER2 CAR T cells, the 4D5 anti-HER2 scFv was ligated into a retroviral backbone vector containing a CD3 zeta chain signaling domain and 4-1BB costimulatory domain. Human peripheral blood mononuclear cells (PBMCs) from healthy donors were activated with anti-CD3/anti-CD28 beads on Day 0 and serially transduced with retrovirus containing the CAR construct on days 2 and 3. Beads were removed on day 4 and T cells were allowed to expand until day 9–11, at which point CAR T cells were harvested. Anti-HER2 CAR expression was assessed by Protein L staining and flow cytometry on Day 4. Representative Protein L staining results are shown for mock transduced T cells, CD19 CAR T cells, and HER2 CAR T cells. **b** HER2 Expression was assessed for the DAOY, D283, and D425 human medulloblastoma cell lines via either qPCR (left) or flow cytometry using the trastuzumab-APC antibody (right). The 928 cell line was used as a positive control for flow cytometry. **c** In a 24-h Incucyte Zoom assay, tumor cells were co-cultured with CD19 CAR or HER2 CAR transduced T cells at the indicated ratios or with trastuzumab. Percent total live tumor cells was measured compared to 100% at time zero. The percentage of live tumor cells at the 24 h time point was compared for each condition using a one-way ANOVA with Sidak’s correction for multiple hypothesis testing. Statistics are shown for the comparison of HER2 CAR 2.5:1 and CD19 CAR 10:1 for all three lines, as well as the comparison of trastuzumab and HER2 CAR 2.5:1 for D425. Denotion of “n.s” indicates a *p* value greater than 0.95. **d** Tumor cells were co-cultured for 24 h with CD19 CAR or HER2 CAR transduced T cells at a 1:1 ratio. IFNγ, IL-2, and TNFα production was measured by a Meso Scale Discovery immunoassay kit, and compared for each condition using multiple T tests with the Holm-Sidak correction

The 4D5 single chain variable fragment (scFv) specific for HER2, derived from the monoclonal antibody trastuzumab, was generously provided by the Surgery Branch at NIH. It was originally cloned into an MSGV (mouse stem cell virus-based splice-gag vector) with a CD3 zeta signaling domain and 4-1BB and CD28 costimulatory motifs. The 4D5 scFv was subcloned into an MSGV with a CD3 zeta signaling domain and 4-1BB costimulatory motif (HER2-BBz CAR) (Fig. 1a). The CD19-BBz-CAR, which utilizes an identical CD3 zeta signaling domain and 4-1BB co-stimulatory motif as the HER2-BBz-CAR, was generated as previously described [18] and was used as controls in all in-vitro and mouse experiments.

### Retrovirus production and transduction of T cells

HER2-BBz-CAR and CD19-BBz-CAR-encoding retroviral supernatants were produced via transient transfection of the 293GP cell line (Clontech). 293GP cells were transfected via Lipofectamine 2000 (Life Technologies) per manufacturer protocols with CAR and RD114 envelope protein encoding plasmids.

Monocyte depleted PBMCs were activated with anti-CD3/CD28 beads (Life Technologies) at a 3:1 bead:cell ratio with 40 IU/ml rh-IL-2 for 3 days. Activated T cells were transduced with retrovirus on days 3 and 4 using Retronectin (Clontech) coated plates, and cultured in 300 IU/ml rh-IL-2. Anti-CD3/CD28 beads were removed on day 5. Media and IL-2 were changed every 2 days. Transduction efficiencies were assessed by flow cytometry [19].

### Flow cytometry

All samples were analyzed with an LSR Fortessa (BD Bioscience) or Gallios 561(Beckman Coulter). Data were analyzed using FlowJo software. CARs were detected with biotinylated protein L (Pierce Protein Biology) followed by streptavidin-conjugated fluorophore. Human T cells obtained from mouse blood and brain were characterized with human antibodies CD45 (HI30, eBioscience), CD4 (OKT4, BioLegend), and CD8 (RPA-T8, eBioscience). Cell line antigen expression was determined with anti-HER2 antibody (HER2Sense™645, red fluorescently labeled trastuzumab).

### Cytotoxicity and cytokine assays

Parental tumor cells were transfected with nuclear locating mCherry (Essen CellPlayer NucLight Red) and antibiotic selected. 5000 target tumor cells were seeded per well in a 96-well plate and co-incubated with CAR T cells or controls for 24 h at effector–to–target ratios ranging from 10:1 to 2.5:1. Cells were cultured at 37°C and 5% CO2 and monitored using an IncuCyte Zoom (Essen BioScience). Images were captured hourly until 8 h and then at 4-h intervals from 4 separate regions per well using a 10X objective. Each experiment was done in triplicate.

Cytokine production by CAR T cells or controls was evaluated by co-incubation with target tumor cells at a 1:1 ratio for 24 h. Supernatants were harvested and cytokine levels measured using a human pro-inflammatory multi-array panel (MesoScale Discovery).

### In-vivo mouse studies

All animal studies were carried out under protocols approved by the NCI Bethesda Animal Care and Use Committee. Xenograft studies were performed using female NSG mice (NOD.Cg-*Prkdc*^*scid*^ *Il2rg*^*tm1Wjl*^/SzJ) from The Jackson Laboratory aged 6–8 weeks with an average weight of 30 g. Mice were anesthetized with 50 mg/kg ketamine and 0.5 mg/kg dexmedetomidine by intraperitoneal (IP) injection. The mice were immobilized in a mouse stereotaxic device (Stoelting). The head was shaved and scrubbed with 1% povidone-iodine, then a 1 cm skin incision was made along the midline and a burr hole was made using an 18G needle. Cerebellar coordinates were − 2 from lambda, + 1 laterally, and 3 mm deep from the surface of the skull. Using a 28G needle mounted on a Hamilton syringe, luciferase expressing Daoy (5 X 10^4^) or D283 (1 X 10^5^) cells in 5 ul were injected through the burr hole over the course of 5 min. The needle was then slowly retracted and the incision closed using 2–3 wound clips. Mice were treated with 1 mg/kg atipamezole IP to reverse the effects of dexmedetomidine. Buprenorphine 0.05 mg/kg was injected subcutaneously for pain control.

For most experiments, mice were treated seven days after tumor implantation and after confirmation of tumor formation by bioluminescence. Mice were distributed randomly across treatment groups. Mice were treated with either 2.5 X 10^6^ T cells intravenously (IV) via tail vein injection or 5 X 10^5^ T cells in 5 ul intratumorally (IT) to the same tumor coordinates. For the experiments to determine the efficacy of treating very established tumors, mice were treated 22 days after tumor implantation and after confirmation of tumor formation by bioluminescence with 5 X 10^6^ T cells IV via tail vein injection. Wound clips were removed 10 days after placement. Peripheral blood was analyzed for T cell persistence. Brains were harvested for additional analysis of T cell persistence after euthanasia at the experimental endpoint. Experimental endpoints were defined by physical signs of distress such as weakness, paralysis, wasting, or significant hair loss during the study or high tumor burden as measured by bioluminescence imaging (greater than 1 X 10^9^ photons/s/cm^2^/sr).

### Bioluminescence imaging

Isofluorane-anesthetized animals were imaged using the IVIS system (IVIS, Xenogen) 10 min after 150 mg/kg D-luciferin (Xenogen) was injected IP. Living Image software was used to quantify the photons emitted from luciferase-expressing cells. Animals were imaged to confirm tumor implantation and then imaged twice weekly.

### Mouse xenograft study design

All animals were treated with T cells after confirmation of tumor engraftment by bioluminescence imaging. For animals that were euthanized to look for human T cells in mouse tissues, the experimental endpoint was approximately 30 days after treatment with T cells. All other xenograft studies were carried out until the previously defined experimental endpoints were reached.

### Non-human primate (NHP) studies

All NHP studies were carried out under protocols approved by the NCI Bethesda Animal Care and Use Committee. Four adult male, healthy, non-tumor bearing rhesus macaques *(Macaca mulatta)* ages 13–15 years, weighing 10.5–14.7 kg, negative for SRV/SIV, and Herpes B viruses were used. The animals were cared for in accordance with the National Research Council (NRC) Guide for the Care and Use of Laboratory Animals. The macaques used all had implanted CNS ventricular and lumbar reservoirs. Animals were sedated (Ketamine, IM, 10 mg/kg, Vedco Inc.) prior to treatment.

### NHP study design

Rhesus PBMCs collected by venipuncture were isolated using a FICOLL gradient. Cells were cultured in AIM-V with 5% FCS and activated with rhesus specific anti-CD3 antibody (BD Bioscience, 20 ng/ml), anti-CD28 antibody (BD Bioscience, 1μg/ml), and 40 IU/ml rh-IL-2. Activated T cells were then transduced with a retrovirus to co-express HER2-BBz-CAR and blue fluorescent protein (BFP) on days 3 and 4 using Retronectin (Takara) coated plates, and cultured in 300 IU/ml recombinant human IL-2. Media and IL-2 were changed every 2 days. Transduction efficiencies were assessed by flow cytometry using both BFP and protein L.

Separately, T cells from NHPs were transduced with a retrovirus (MSGV) encoding truncated HER2 and GFP to create autologous cells with human HER2 surface expression (T-HER2). Transduction efficiencies were assessed by flow cytometry using both GFP and anti-HER2 antibodies.

1.5 X 10^5^ autologous T-HER2 cells in 0.5 ml sterile PBS were instilled over 1 min into a lateral ventricle reservoir in each NHP. Thirty minutes later, all animals were treated with 1.5 X 10^5^ autologous HER2-CAR T cells in 0.5 ml sterile PBS over 1 min into the same ventricular reservoir. Cerebrospinal fluid (CSF) was collected 1, 3, and 7 days after treatment through a lumbar reservoir. Peripheral blood was collected at the same time-points. CSF and blood were analyzed by flow cytometry for persistence of both T-HER2 and HER2-CAR T cells. CSF and plasma were analyzed for cytokines using the NHP Proinflammatory Panel 1 kit (MesoScale Discovery).

### Statistics

Prism 6 software was utilized for statistical analysis. Cytokine concentrations and immune cytotoxicity were analyzed using a one-way ANOVA with Bonferroni’s correction for multiple comparisons. Comparison of T cells found in mouse blood/tissues across groups was done with using the Mann-Whitney nonparametric unpaired U-test. Xenograft growth curves were compared using Living Image software to quantify tumor size for individual mice and then analyzed. Luminescence values at each time point were converted to log form and linear regression was used to find a curve of best fit for each treatment group. The slopes of these curves were then compared in a pairwise fashion to look for differences in the overall rate of tumor growth [20].

## Results

### HER2-BBz-CAR T cells kill medulloblastoma cells in-vitro and produce effector cytokines

Medulloblastoma cell lines Daoy, D283, and D425 were examined for HER2 expression by flow cytometry. Daoy and D283 both exhibited HER2 surface expression, while the D425 cell line exhibited no expression of HER2 (Fig. 1b) and served as a negative control. Ependymoma 928 cell line exhibited HER2 surface expression and served as a positive control. Second generation CARs contain a single co-stimulatory domain in addition to the CD3 zeta signaling domain. We chose to utilize the 4-1BB (CD137) co-stimulatory domain, as previous work has shown it to be superior in reducing T cell exhaustion and enhancing T cell persistence compared to CD28 [19, 21]. HER2-BBz-CAR and CD19-BBZ-CAR T cells were generated with transduction efficiencies between 40 and 80% (Fig. 1a). Additional characterization of HER2-BBz-CAR T cell subtype, as well as activation and exhaustion markers are displayed in Additional file 1: Figure S1. There was no significant difference in T cell phenotype when comparing HER2-BBZ-CAR T cells to non-transduced, activated T cells, designated as mock.

Using CD19-BBz-CAR T cells and D425 cells as negative controls, HER2-CAR T cell cytotoxicity towards Daoy, D283, and D425 was assessed using an Incucyte Zoom assay over a 24-h period at an effector to target (E:T) ratio of 10:1 to 2.5:1. HER2-BBz-CAR T cells demonstrated significant killing of Daoy and D283, not D425, even at low E:T ratios, and there was no cytotoxicity observed from either CD19-BBZ CAR T cells or trastuzumab (Fig. 1d), which is consistent with prior reports [11]. This data suggests that the HER2-BBz-CAR confers specific killing activity against HER2-positive medulloblastomas.

To assess cytokine production by T cells, we performed a 24-h co-culture assay of HER2-BBz-CAR T cells or CD19-BBz-CAR T cells with either Daoy, D283, or D425 cells at an E:T ratio of 1:1. Supernatants from these cultures were then analyzed for IFNγ, IL-2, and TNFα concentration by multiplex array. HER2-BBz-CAR T cells produced significantly higher levels of IFNγ, IL-2, and TNFα in response to Daoy and D283 compared to CD19-BBz-CAR T cells. Additionally, HER2-BBz-CAR T cells did not produce significant levels of any cytokine when co-incubated with D425 medulloblastoma cells (Fig. 1e). These findings support specific HER2 antigen recognition by and activation of HER2-BBz-CAR T cells in the presence of HER2-bearing medulloblastomas.

### Intratumorally delivered HER2-BBz-CAR T cells eliminate medulloblastoma in murine xenografts

**Fig. 2:**
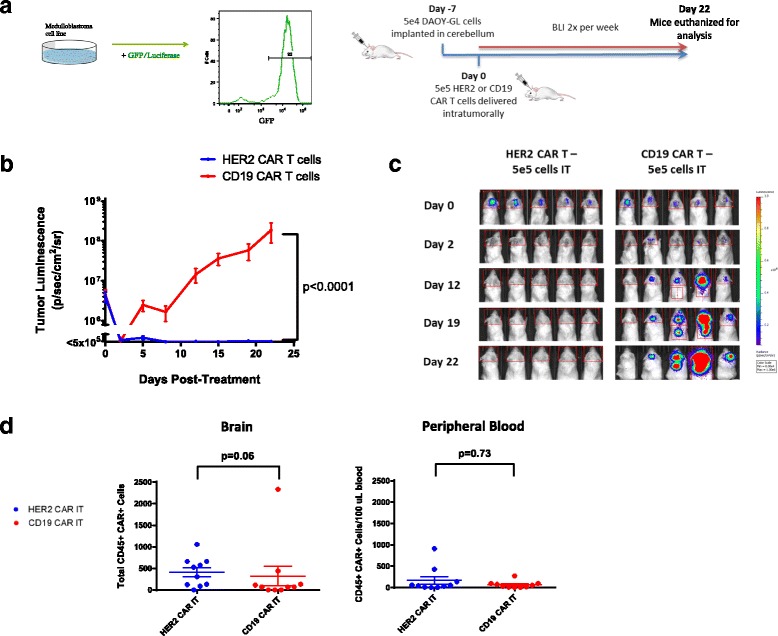
Intratumoral administration of HER2 CAR T cells effectively treats medulloblastoma in an orthotopic model. **a** Outline of experimental setup. DAOY human medulloblastoma cells were transduced with lentivirus containing a GFP-Luciferase construct to facilitate in vivo study. Example DAOY-GL GFP expression is shown by flow cytometry. For the experiments, NOD.Cg-Prkdcscid Il2rgtm1Wjl/SzJ (NSG) mice were given tumor and treated with human CAR T cells intratumorally (IT) as shown. Bioluminescent imaging was performed using a Xenogen IVIS apparatus twice per week. **b** Tumor growth curves for NSG mice treated with either HER2 and CD19 CAR T cells intratumorally on Day 0. Results are shown for two experiments using T cells from two separate donors. Indicated doses correspond to the number of human CAR+ T cells infused into each animal. Error bars represent standard error of the mean. For shown statistics, data were normalized, linear regressions fitted to the normalized data, and slopes were compared using an ANOVA test. **c** Bioluminescent images from selected timepoints during the experiments. Indicated doses represent number of CAR positive T cells injected per mouse. **d** Brain and peripheral blood were harvested from mice on day 22 post- treatment and analyzed by flow cytometry. The presence of human CAR+ T cells was assessed by CD45 and Protein-L staining. Total amounts of human CAR T cells in the brain and relative concentrations in the peripheral blood for each group were compared using Mann-Whitney non-parametric statistical tests

To create our mouse xenografts, Daoy cells were stably transduced with a GFP-luciferase lentiviral construct (Daoy-GL) to allow for the tracking of tumor growth over time via bioluminescence (Fig. 2a). Daoy-GL cells were flow sorted and then single cell cloned to ensure purity of the transduced population. 5 X 10^4^ Daoy-GL cells were injected into the cerebella of NOD.Cg-*Prkdc*^*scid*^ *Il2rg*^*tm1Wjl*^/SzJ (NSG) immunodeficient mice and allowed to grow for one week (Fig. 2a) before T cell treatment.

Impaired CAR T cell trafficking from the blood to tumor is one of the major limitations of CAR T cell therapy in solid tumors [22]. We hypothesized that administering CAR T cells directly to the tumor site would eliminate this issue and would require significantly fewer CAR T cells to achieve the same response as intravenously administered CAR T cells. This approach has been used previously with limited success [11], albeit with a first-generation CAR. 5 X 10^5^ human HER2-BBz-CAR T cells or CD19-BBz-CAR T cells were delivered IT 7 days following tumor cell injection, after which tumor growth was followed via bioluminescent imaging (Fig. 2b; Additional file 3: Figure S3a).

As predicted, mice treated with IT-delivered HER2-BBz-CAR T cells experienced complete tumor regression within 7–10 days and did not relapse for the duration of the experiments. By contrast, mice treated with IT-delivered CD19-BBz-CAR T cells experienced rapid tumor progression requiring euthanasia at approximately four weeks (Fig. 2b and c) (*n* = 20) and had evidence of tumor upon immunohistochemical examination of the brain post-mortem (Additional file 2: Figure S2A).

### Regional delivery of HER2-BBz-CAR T cells is more efficient in medulloblastoma xenografts than intravenous treatment

**Fig. 3:**
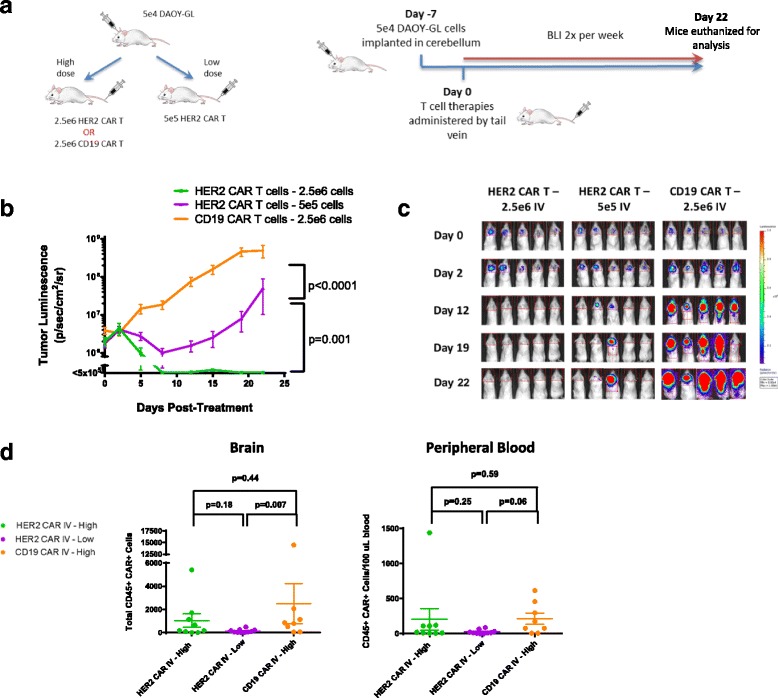
Intravenous HER2 CAR T cells can effectively treat medulloblastoma, but requires a higher dose level. **a** Outline of experimental setup. NOD.Cg-Prkdcscid Il2rgtm1Wjl/SzJ (NSG) mice were given tumor and treated with human CAR T cells IV as shown, at one of two dose levels. Bioluminescent imaging was performed using a Xenogen IVIS apparatus twice per week. **b** Tumor growth curves for NSG mice treated with either HER2 and CD19 CAR T cells IV at both high and low dose levels on Day 0. Results are shown for two experiments using T cells from two separate donors. Error bars represent standard error of the mean. For shown statistics, linear regressions were fitted to each curve and slopes were compared using an ANOVA test. **c** Bioluminescent images from selected timepoints during the experiments. Indicated doses represent number of CAR positive T cells injected per mouse. **d** Brain and peripheral blood were harvested from mice on day 22 post- treatment and analyzed by flow cytometry. Total amounts of human CAR T cells in the brain and relative concentrations in the peripheral blood were measured and compared as previously described in Fig. 2

We next sought to examine the efficacy of IV delivery of HER2-BBz-CAR T cells in medulloblastoma-bearing mice. After establishing Daoy-GL tumors as before, either 5 X 10^5^ or 2.5 X 10^6^ HER2-BBz-CAR T cells or 2.5 X 10^6^ CD19-BBz-CAR T cells were injected IV and mice were followed by bioluminescence (Fig. 3a).

Following an initial, short-term regression, tumors progressed in mice treated intravenously with 5 X 10^5^ HER2-BBz-CAR T cells (Fig. 3b and c). This is in contrast to the complete resolution of tumors in mice treated with the same dose IT (Fig. 2b; Additional file 3: Figure S3a). In order to produce complete regression of tumor, a five-fold higher cell dose (2.5 X 10^6^) was required when given IV versus IT. CD19-BBz-CAR T cells had no significant effect on tumor growth (Fig. 3b and c) (*n* = 30) and euthanized mice had evidence of tumor on histology (Additional file 2: Figure S2B).

These results demonstrate that IV administration of HER2-BBz-CAR T cells at the same cell dose is less effective at controlling tumor growth than IT administration and that higher doses of CAR T cells are required for an equivalent level of disease control when given IV.

### HER2-BBz-CAR T cells eliminate medulloblastoma in a high-risk murine xenograft model

**Fig. 4:**
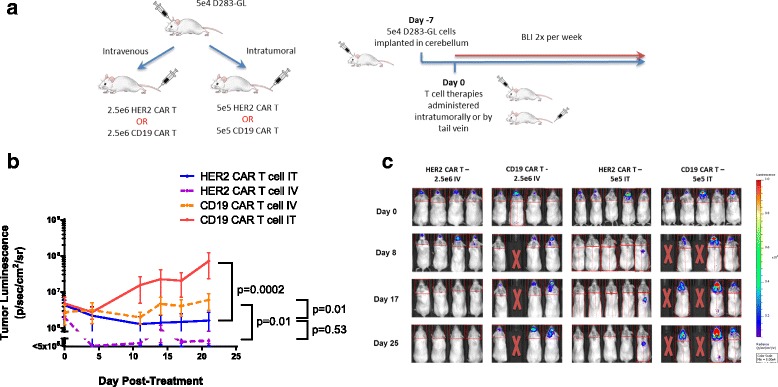
HER2 CAR T cells can effectively treat medulloblastoma in a second orthotopic model. **a** Outline of experimental setup. NOD.Cg-Prkdcscid Il2rgtm1Wjl/SzJ (NSG) mice were given tumor and treated with human CAR T cells IV or IT as shown, at one of two dose levels. Bioluminescent imaging was performed using a Xenogen IVIS apparatus twice per week. **b** Tumor growth curves for NSG mice with previously described treatments on Day 0. Error bars represent standard error of the mean. For shown statistics, linear regressions were fitted to each curve and slopes were compared using an ANOVA test. **c** BLI images of mice from each group at selected timepoints. Indicated doses represent number of CAR positive T cells injected per mouse

To explore the broad applicability of our observations in medulloblastoma, we established a second xenograft mouse model using the D283 cell line. This cell line shows characteristics of the Group 3 and 4 subgroups of medulloblastoma, which have a worse prognosis than the Sonic Hedgehog (SHH) subgroup represented by the Daoy cell line [23]. D283 cells were stably modified with GFP-luciferase (D283-GL). We injected 5 X 10^4^ D283-GL into the cerebella of NSG mice as previously described, and tumor engraftment was confirmed by bioluminescent imaging at one week. HER2-BBz or CD19-BBz-CAR T cells were then delivered either IT (5 X 10^5^ CAR T cells) or IV (2.5 X 10^6^ CAR T cells) (Fig. 4a) (*n* = 36).

IT delivered HER2-BBZ-CAR T cells at this very low dose produced complete regression of the majority of tumors, similar to the effect seen in the Daoy-GL model (Fig. 4b and c; Additional file 3: Figure S3c). IV delivery of HER2-BBz-CAR T cells at the dose that produced complete Daoy-GL tumor regression (Fig. 3b; Additional file 3: Figure S3b) was sufficient to eliminate D283-GL tumors, and both IT- and IV-delivered HER2-BBz-CAR T cells were significantly more effective than CD19-CAR T cells (Fig. 4b and c; Additional file 3: Figure S3c). Together, these observations in Daoy and D283 xenograft models suggest that HER2-BBz-CAR T cells are likely to be effective across a broad array of medulloblastomas with HER2 surface expression, regardless of their molecular subgroup.

### HER2-BBz-CAR T cells eliminate well-established medulloblastoma in a murine xenograft model

**Fig. 5:**
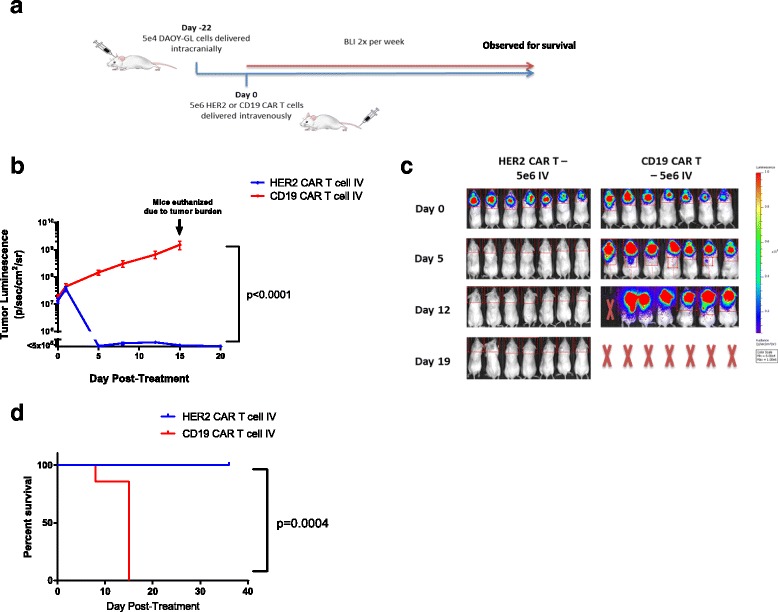
HER2 CAR T cells can effectively treat large, established medulloblastoma xenografts. **a** Outline of experimental setup. NOD.Cg-Prkdcscid Il2rgtm1Wjl/SzJ (NSG) mice were given tumor on Day 0 and tumor were allowed to grow until day 22. Mice were treated with 5e6 HER2-BBz CAR T cells or CD19 CAR T cells on Day 22 and tumor growth was monitored by regular BLI imaging for approximately five weeks. Remaining mice were then placed under long-term observation for tumor recurrence. Bioluminescent imaging was performed using a Xenogen IVIS apparatus. **b** Tumor growth curves for NSG mice with previously described treatments applied on Day 0. Error bars represent standard error of the mean. For statistics, linear regressions were fitted to each curve and slopes were compared using an ANOVA test. **c** BLI images of mice from each group at selected timepoints. Indicated doses represent number of CAR positive T cells injected per mouse. **d** Overall survival of mice from each group. HER2 CAR T cell-treated mice were euthanized on Day 37 post-treatment due to animal protocol restrictions, but had no observable tumors (data not shown)

After observing the potent anti-tumor effects of HER2-BBz-CAR T cells on medulloblastoma xenografts in previous experiments, we wanted to test whether or not these cells could produce responses in larger, more established tumors. To do so, we delayed CAR T cell treatment from 7 days after tumor cell injection to 22 days. Between these time-points, tumor luminescence was regularly monitored and treatment was started when the average tumor size of each group of mice approached 2 X 10^7^ p/s/cm^2^/sr or approximately 10-fold higher than previous experiments. Since the burr hole created to deliver tumor cells had closed by this point, CAR T cells were administered by IV route only. To compensate for the higher initial tumor burden, a higher dose of 5 X 10^6^ HER2-BBz or CD19-BBz-CAR T cells were administered IV and tumor size post-treatment was measured twice weekly by bioluminescence (Fig. 5a).

As expected, mice given CD19-BBz-CAR T cells experienced rapid tumor progression and had to be euthanized due to excessive tumor burden 15 days following treatment (37 days after tumor implantation). In contrast, mice given HER2-BBz-CAR T cells experienced rapid tumor regression within 1 to 5 days, with complete regression of all tumors observed by day 5 (Fig. 5b and c; Additional file 3: Figure S3d) (*n* = 14). The increased potency of this effect in comparison to the other experiments is likely explained by the increased dose of T cells administered, rather than a biological effect unique to the higher tumor burden setting. These mice remained healthy and clear of tumor during regular follow-up, for a total of 36 days post-treatment, and had a significantly prolonged survival (Fig. 5d). Mice were euthanized due to progressive signs of xenogeneic graft versus host disease, a known complication of using human CAR T cells in murine xenografts [24]. These results further strengthen our previous findings that treatment with HER2-BBz-CAR T cells leads to complete regression of orthotopic medulloblastoma, including very established tumors.

### HER2-BBz-CAR T cells persist at low levels in the brain and peripheral blood

After observing the effect of HER2-BBz-CAR T cells on established medulloblastoma xenografts, we wanted to determine whether or not these T cells are capable of persistence in these animals. Thirty days after CAR T cell injection (IV or IT), mice were euthanized and brain and peripheral blood were obtained. Single cell suspensions were generated from the brain and peripheral blood of each mouse and analyzed by flow cytometry for the human lymphocyte markers CD45, CD4 and CD8, as well as Protein L to detect expression of the CAR.

We found persistence of CAR T cells in the blood and brain of mice treated regionally and intravenously with CAR T cells (Figs. 2d and 3d). We found that there was a trend towards greater persistence of HER2-BBz CAR-T cells in the brain when administered intratumorally as compared to CD19-BBz CAR-T cells, but overall there were no statistically significant differences. Higher dose levels of CAR-T cells administered intravenously also seemed to be associated with greater persistence in both the brain and peripheral blood, but this effect was only significant in the case of CD19-BBz CAR T cells. Most likely, an immunocompromised, lymphodeplete NSG mouse is simply not an appropriate model to differentiate persistence between different CAR T cells and the routes of administration since unmodified human T cells engraft in NSG mice regardless of the presence of a target antigen [24].

### HER2-BBz-CAR T cells can be safely administered via an intraventricular route in non-human primates (NHPs)

Given the concern for on-target, off-tumor systemic toxicity of HER2-directed CAR T cells [25] and the propensity for medulloblastoma to metastasize along the leptomeninges of the brain and spinal cord, intraventricular delivery of HER2-BBz-CAR T cells may be preferred over IV administration. To elucidate the kinetics and compartmentalization resulting from intraventricular HER2-BBz-CAR T cell delivery, NHPs bearing lateral ventricle and lumbar reservoirs were employed. These animals are excellent models for anti-HER2 therapeutic testing due to the fact that rhesus macaques have 98% homology to human receptor tyrosine protein kinase ERBB2 [26], and trastuzumab, the monoclonal antibody that the HER2-BBZ-CAR T cell derives its specificity from, recognizes a receptor on NHP epithelial cells [27].

**Fig. 6:**
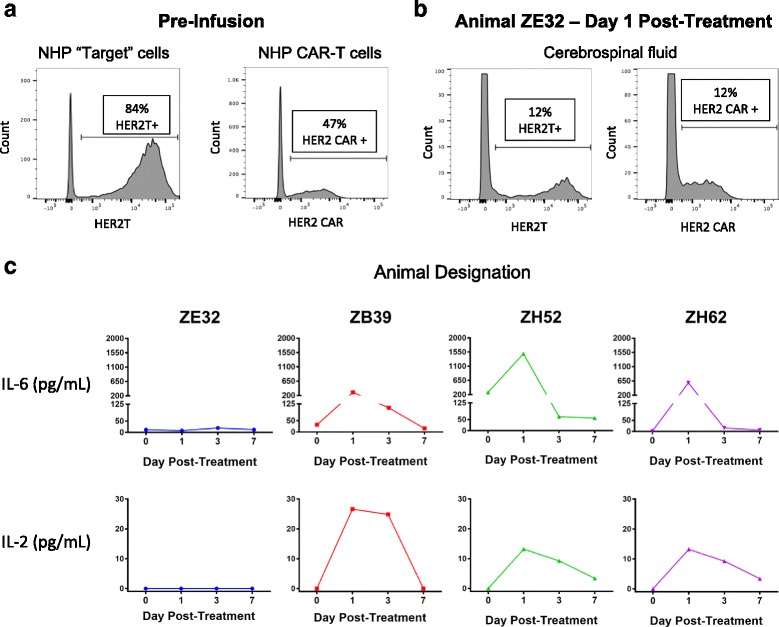
HER2-CAR T cells administered intrathecally to non-human primates (NHP) did not cause toxicity. **a** Flow cytometry of truncated HER2 expression (left) and HER2 CAR expression (right) in NHP T cells prior to infusion. The transduction efficiency for truncated HER2 and HER2 CAR ranged from 45 to 90% in all cell cultures. **b** Flow cytometry of truncated HER2 expression (left) and HER2 CAR expression (right) in T cells from the cerebrospinal fluid (CSF) of NHP ZE32 on Day 1 after infusion. T-HER2 and HER2 CAR T cells were not detected in the other three animals, nor were appreciable levels detected after Day 1 in animal ZE32. **c** Summary of CSF cytokine levels after infusion of T-HER2 target cells and HER2 CAR T cells. Separate time courses of cytokine measurements, performed by ELISA, are shown for each animal in the experiment. Increases in IL-2 and IL-6 were detected in the CSF of most animals, suggesting antigen recognition of the truncated HER2 protein by the HER2 CAR T cells. IL-8, IL-10, and IL-1β were also tested for, but were undetectable in all samples

Autologous rhesus T cells modified to express truncated HER2 (T-HER2) were administered intraventricularly to serve as a target for HER2-BBz-CAR T cells (*n* = 4). Transduction efficiencies for T-HER2 and HER2-BBz-CAR T cells ranged from 45 to 90% (Fig. 6a). 1.5 X 10^5^ T-HER2 cells were infused into a ventricular reservoir and followed thirty minutes later by the infusion of 1.5 X 10^5^ autologous HER2-BBz-CAR rhesus T cells. All animals had blood counts and chemistries at baseline and then every other day; all values were within normal limits. A standardized neurological assessment was performed at baseline and then daily, and subjects showed no changes from baseline. CSF from the lumbar reservoir and plasma were sampled prior to cell delivery and 1, 3, and 7 days later. T cells expressing T-HER2 and HER2-BBz-CAR were detected in the CSF of one animal 24 h after treatment (Fig. 6b). Increases in IL-6 and IL-2 were detected in the CSF of most animals (Fig. 6c). Increased IL-2 suggests antigen recognition of the truncated HER2 protein by the HER2-BBz-CAR T cells. No T cells expressing truncated HER2 or HER2-BBz-CAR and no elevated cytokines were detected in the blood or plasma of treated animals (data not shown).

## Discussion

Prior pre-clinical studies of HER2-directed CAR T cells for medulloblastoma had limited efficacy primarily due to reliance on first-generation, CD3 zeta constructs [11]. Multiple second-generation CD19 CAR clinical trials utilizing 41BB or CD28 co-stimulatory domains with CD3 zeta have resulted in excellent clinical responses in patients with B cell malignancies [13, 14, 28–33]; 41BB co-stimulation results in longer persistence of CAR T cells in some patients and murine studies directly comparing 41BB and CD28 co-stimulatory domains demonstrate this [18]. A Phase I clinical trial of intravenously administered HER2-CD28-CD3 zeta CAR modified virus-specific T cells in patients with glioblastoma showed few and limited responses [17]. We investigated the efficacy of targeting HER2-expressing medulloblastoma using a modern CAR T cell via both IV and regional routes in mice. We chose a second-generation construct utilizing 4-1BB co-stimulation because of its proven improved persistence in clinical trials and published literature showing potent T cell activation and decreased, potential for T cell exhaustion [19].

We showed that human HER2-BBz-CAR T cells could eliminate human medulloblastoma cells both in-vitro and in mouse models of medulloblastoma. We also demonstrated that HER2-BBz-CAR T cells produce significant levels of inflammatory cytokines during co-culture with medulloblastoma, which is a key determinant of a successful anti-tumor T cell response in humans [34]. Furthermore, we found that HER2-BBz-CAR T cells could fully eliminate even very large, established tumors. Together, these data indicate that HER2-BBz-CAR T cells possess highly potent anti-tumor activity in medulloblastoma.

Tumor responses from HER2-BBz-CAR T cells were observed regardless of the route of administration, though a log fewer CAR T cells were required to achieve the same effect when given regionally versus IV. Successful regional delivery of a lower but equally as effective CAR T cell dose may mitigate on-target, off-tumor systemic toxicity and/or cytokine release syndrome [35]. This route is potentially beneficial for patients with disease localized to one compartment.

To evaluate the feasibility of intraventricular CAR T cell delivery, and explore whether this route would result in systemic toxicity, we utilized NHPs with existing ventricular and lumbar reservoirs. Since tumor lines cannot be introduced to these animals, we chose to modify rhesus autologous T cells with the extracellular domain of HER2 to serve as targets of the CAR T cells. All but one animal displayed an elevation of IL-6 in the CSF after infusion of autologous T cells. IL-6 is not produced directly by the CAR T cell in-vitro (data not shown) but rather directly by or after interaction with endogenous antigen presenting cells [36], suggesting recruitment of the NHP’s immune system after CAR T cell infusion. Elevated IL-2 was measured in 3 out of 4 animals, which also suggests recognition of T-HER2 cells by HER2-CAR T cells. Interestingly, only one animal had persistence of T-HER2 and HER2-CAR T cells at 24 h and no elevation of IL-2, which suggests a failure of the HER2-CAR T cells to recognize the T-HER2 targets in this animal. Importantly, no systemic exposure of HER2-BBz-CAR T cells was found in any animal and plasma showed no significantly elevated inflammatory cytokines. We therefore conclude that intraventricular delivery of HER2-BBz-CAR T cells is feasible and, at the doses studied, did not result in significant systemic exposure or toxicity.

There are limitations to our study that bear discussion. Though the NSG mouse model is an excellent pre-clinical tool for assessing the interactions between human tumor cells and human T cells, it does not capture the interaction of these cells in the context of the human immune system or tumor microenvironment. Second, we utilized medulloblastoma cell lines instead of patient derived tumor xenografts. We made this choice due to the greater flexibility allowed in working with cell cultures to perform correlative in-vitro assays and the ability to transduce cell lines with GFP and luciferase to facilitate in-vivo identification. Though these cell lines may be less representative of the tumor biology seen in patients, a key advantage of CAR T cell therapy efficacy is that it primarily requires only that the target of the CAR be expressed on the cell surface, meaning that cell line models can be more appropriate for testing these types of therapies than in other cases.

A third limitation of our study surrounds the blood-brain-barrier (BBB) in mice and the propensity of T cells to cross that barrier. Sampson, et al. demonstrated that IV administered murine EGFRvIII-CAR T cells can effectively clear orthotopic murine glioblastoma in the forebrain of immunocompetent mice [37]. However, another group showed only a reduction rather than clearance of human glioblastoma tumors following IV administration of human EGFRvIII-CAR T cells [38]. This is consistent with an earlier study showing the failure of IV-delivered, second generation human EphA2-CAR T cells to cause any tumor regression of human glioma orthotopically implanted in the brain of SCID (severe combined immunodeficiency) mice [39]. A recent publication of a human IL13rα2-CAR T cell only studied regional delivery in an orthotopic human glioma mouse model, citing the previous failures of IV administered cells in clearing tumors in mouse brain, hypothesizing that these failures were due to the inability of human T cells to cross the murine BBB [40].

In our study, IV-administered HER2-BBz-CAR T cells in sufficient doses are effective at eliminating stereotactically implanted medulloblastoma in the posterior fossa of xenografts. The initial injection of tumor cells into the brain certainly disrupts the BBB, which may permit translocation of CAR T cells several days later. However, it is possible that CAR T cells are not impeded by the BBB in any system. A functional lymphatic system in the CNS has been described in mice [41] and, in a pivotal Phase I study of CD19-directed CAR T cells in children and young adults with acute lymphoblastic leukemia, two patients with CNS leukemia had complete responses in the CSF from a single IV dose of CAR T cells [13]. So, while others have shown limited efficacy of IV dosing in animals, IV administration of HER2-BBz CAR T cells for medulloblastoma may be effective and could be explored in human trials should intraventricular delivery prove not to be effective.

The principal concern of systemically-administered HER2-CAR T cells stems from potential on-target but off-tumor cytotoxicity, especially in vital organs. A third generation HER2-CAR T cell trial was stopped after the first patient died within days after infusion of cells in 2010 [25]. It was initially hypothesized that the cause of death was due to low levels of HER2 expression found on heart and lung, but the safety and tolerability demonstrated in newer HER2-directed CAR T cell trials would seem to refute this hypothesis. HER2 has been targeted with second generation CAR T cell therapies in patients with sarcoma and glioblastoma without significant toxicity [16, 17]. The biggest differences between these studies were dose of CAR T cells infused, generation of CAR employed, the use of a high-dose lymphodepleting preparative regimen, and concurrent high-dose of IL-2 given with the cell infusion [25]. Cytokine release syndrome (CRS), a known life-threatening complication of adoptive cell therapy resulting from the excessive release of inflammatory cytokines by supra-physiologically activated immune cells, can unfold within hours to days after cell infusion, consistent with the rapid onset of life-threatening symptoms observed in the 2010 patient [42]. Our understanding of the pathophysiology and management of CRS has increased significantly since this incident as more patients have been treated with adoptive cell therapies [42]. Careful consideration of dosing and lymphodepleting regimens coupled with timely intervention with anti-cytokine drugs have made CAR T cell therapy safer and more feasible for clinical investigation.

## Conclusions

Children and adults with relapsed medulloblastoma have a dismal prognosis even with aggressive therapy. Given our data, advances in the understanding of CAR T cell therapies, and the recently published case report of one patient who safely received intraventricular IL13Rα2-targeted CAR T cells [43], intraventricular delivery of HER2-BBz-CAR T cells starting at a low-dose using careful dose-escalation without a systemic lymphodepleting regimen is a rational approach to translate this promising therapy to patients with relapsed medulloblastoma.

## Additional files

Additional file 1: **Figure S1.** Characterization of HER2 CAR T cell phenotype. Human T cells were either mock transduced or transduced with HER2 CAR as previously described in Methods. Cells were taken from culture on Day 7 and stained for CD4, CD8, and either a panel of markers of T cell activation (A) or a panel of markers of T cell exhaustion (B). CAR+ T cells were identified by Protein-L staining as previously shown in Fig. 1. Histograms for each population of T cells are shown above, as well as the relevant FMO controls. (PPTX 389 kb)

Additional file 2: **Figure S2.** Medulloblastoma xenograft histology. NOD.Cg-Prkdcscid Il2rgtm1Wjl/SzJ (NSG) mice were injected with DAOY-GL tumor cells, as described in Methods, and treated with human CD19 CAR T cells intratumorally (A) or intravenously (B). Mice were euthanized at day 22 post-treatment and brain tissue was collected for histology. Brains were sectioned and stained using H&E. Images were taken using a digital slide scanner at 10X magnification, with representative results shown above. DAOY-GL cells mainly formed tumors along the periphery of the cerebellum (indicated by black arrows), but can also be seen infiltrating into the parenchyma adjacent to normal cerebellar cells (indicated by red arrow). (PPTX 5146 kb)

Additional file 3: **Figure S3.** Linear regression data used for calculating statistics from Figs. 2, 3, 4, and 5. Data is presented as spider plots, with each line representing data from an individual mouse, and linear regression lines and equations overlaid. Fig. 2b. Fig. 3b. Fig. 4b. Fig. 5b. (PPTX 274 kb)

## Abbreviations

ATCC: American Type Culture Collection
BBB: blood-brain-barrier
CAR: chimeric antigen receptor
CRS: cytokine release syndrome
CSF: cerebrospinal fluid
E:T: effector to target
FCS: fetal calf serum
GFP: green fluorescent protein
HER2: human epidermal growth factor receptor 2
IP: intraperitoneal
IT: intratumorally
IV: intravenously
MSGV: (mouse stem cell virus-based splice-gag vector)
NHP: non-human primate
NSG: NOD.Cg-*Prkdc*^*scid*^ *Il2rg*^*tm1Wjl*^/SzJ
PBMC: peripheral blood mononuclear cell
PSG: penicillin-streptomycin-glutamine
scFv: single chain variable fragment
T-HER2: truncated HER2

## References

[1] Martin AM (2014). Management of Pediatric and Adult Patients with Medulloblastoma. Curr Treat Options in Oncol.

[2] Packer RJ (2013). Survival and secondary tumors in children with medulloblastoma receiving radiotherapy and adjuvant chemotherapy: results of Children's oncology group trial A9961. Neuro-Oncology.

[3] Fouladi M (2013). A molecular biology and phase II trial of lapatinib in children with refractory CNS malignancies: a pediatric brain tumor consortium study. J Neuro-Oncol.

[4] Orentas RJ, Lee DW, Mackall C (2012). Immunotherapy targets in pediatric cancer. Front Pediatr Oncol.

[5] Fousek K, Ahmed N (2015). The evolution of T-cell therapies for solid malignancies. Clin Cancer Res.

[6] Ahmed N (2010). HER2-specific T cells target primary glioblastoma stem cells and induce regression of autologous experimental tumors. Clin Cancer Res.

[7] Ahmed N (2009). Immunotherapy for osteosarcoma: genetic modification of T cells overcomes low levels of tumor antigen expression. Mol Ther.

[8] Gilbertson RJ (1992). Originally published as volume 2, issue 8817Prognostic factors in medulloblastoma. Lancet.

[9] Gilbertson RJ (1995). Prognostic significance of the c-erbB-2 oncogene product in childhood medulloblastoma. Br J Cancer.

[10] Uhlén M, et al. Tissue-based map of the human proteome. Science. 2015;347(6220) 10.1126/science.1260419 25613900

[11] Ahmed N (2007). Regression of experimental Medulloblastoma following transfer of HER2-specific T cells. Cancer Res.

[12] Lee DW (2012). The future is now: chimeric antigen receptors as new targeted therapies for childhood Cancer. Clin Cancer Res.

[13] Lee DW (2015). T cells expressing CD19 chimeric antigen receptors for acute lymphoblastic leukaemia in children and young adults: a phase 1 dose-escalation trial. Lancet.

[14] Maude SL (2014). Chimeric antigen receptor T cells for sustained remissions in leukemia. N Engl J Med.

[15] Liu D (2013). Medulloblastoma expresses CD1d and can be targeted for immunotherapy with NKT cells. Clin Immunol.

[16] Ahmed N (2015). Human epidermal growth factor receptor 2 (HER2) –specific chimeric antigen receptor–modified T cells for the immunotherapy of HER2-positive sarcoma. J Clin Oncol.

[17] Ahmed N (2017). Her2-specific chimeric antigen receptor–modified virus-specific t cells for progressive glioblastoma: a phase 1 dose-escalation trial. JAMA Oncol..

[18] Milone MC (2009). Chimeric receptors containing CD137 signal transduction domains mediate enhanced survival of T cells and increased Antileukemic efficacy in vivo. Mol Ther.

[19] Long AH (2015). 4-1BB costimulation ameliorates T cell exhaustion induced by tonic signaling of chimeric antigen receptors. Nat Med.

[20] Heitjan DF, Manni A, Santen RJ (1993). Statistical analysis of in vivo tumor growth experiments. Cancer Res.

[21] Campana D, Schwarz H, Imai C. 4-1BB chimeric antigen receptors. The Cancer Journal. 2014;20(2) 10.1097/PPO.0000000000000028 24667959

[22] Beatty GL, O'Hara M (2016). Chimeric antigen receptor-modified T cells for the treatment of solid tumors: defining the challenges and next steps. Pharmacol Ther.

[23] Liang L (2015). Characterization of novel biomarkers in selecting for subtype specific medulloblastoma phenotypes. Oncotarget.

[24] Ali N (2012). Xenogeneic graft-versus-host-disease in NOD-scid IL-2Rγ(null) mice display a T-effector memory phenotype. PLoS One.

[25] Morgan RA (2010). Case report of a serious adverse event following the Administration of T Cells Transduced with a chimeric antigen receptor recognizing ERBB2. Mol Ther.

[26] Deng X (2014). Comparative analysis of evolutionarily conserved motifs of epidermal growth factor receptor 2 (HER2) predicts novel potential therapeutic epitopes. PLoS One.

[27] Herceptin: EPAR-Scientific Discussion. 2005, European Medicines Agency.

[28] Brentjens RJ (2013). CD19-Targeted T Cells Rapidly Induce Molecular Remissions in Adults with Chemotherapy-Refractory Acute Lymphoblastic Leukemia. Sci Transl Med.

[29] Kalos M (2011). T cells with chimeric antigen receptors have potent antitumor effects and can establish memory in patients with advanced leukemia. Sci Transl Med.

[30] Kochenderfer JN (2012). B-cell depletion and remissions of malignancy along with cytokine-associated toxicity in a clinical trial of anti-CD19 chimeric-antigen-receptor–transduced T cells. Blood.

[31] Kochenderfer JN, et al. Chemotherapy-refractory diffuse large B-cell lymphoma and indolent B-cell malignancies can be effectively treated with autologous T cells expressing an anti-CD19 chimeric antigen receptor. J Clin Oncol. 2015;33 10.1200/JCO.2014.56.2025 PMC4322257 25154820

[32] Grupp SA (2013). Chimeric antigen receptor–modified T cells for acute lymphoid leukemia. N Engl J Med.

[33] Davila ML, et al. Efficacy and toxicity management of 19-28z CAR T cell therapy in B cell acute lymphoblastic leukemia. Sci Transl Med. 2014;6 10.1126/scitranslmed.3008226 PMC4684949 24553386

[34] Jensen MC, Riddell SR (2014). Design and implementation of adoptive therapy with chimeric antigen receptor-modified T cells. Immunol Rev.

[35] Brudno JN, Kochenderfer JN (2016). Toxicities of chimeric antigen receptor T cells: recognition and management. Blood.

[36] Barrett DM (2016). Interleukin 6 is not made by chimeric antigen receptor T cells and does not impact their function. Blood.

[37] Sampson JH (2014). EGFRvIII mCAR-modified T-cell therapy cures mice with established intracerebral glioma and generates host immunity against tumor-antigen loss. Clin Cancer Res.

[38] Johnson LA (2015). Rational development and characterization of humanized anti–EGFR variant III chimeric antigen receptor T cells for glioblastoma. Sci Transl Med.

[39] Chow KKH (2013). T cells redirected to EphA2 for the immunotherapy of glioblastoma. Mol Ther.

[40] Krenciute G (2016). Characterization and functional analysis of scFv-based chimeric antigen receptors to redirect T cells to IL13R[alpha]2-positive glioma. Mol Ther.

[41] Louveau A (2015). Structural and functional features of central nervous system lymphatic vessels. Nature.

[42] Lee DW (2014). Current concepts in the diagnosis and management of cytokine release syndrome. Blood.

[43] Brown CE (2016). Regression of glioblastoma after chimeric antigen receptor T-cell therapy. N Engl J Med.

