# Supplementary figures and images for: Durable regression of Medulloblastoma after regional and intravenous delivery of anti-HER2 chimeric antigen receptor T cells
Source: J Immunother Cancer. 2018 Apr 30;6:30. doi: 10.1186/s40425-018-0340-z (PMC5925833; doi:10.1186/s40425-018-0340-z)

## Slide 1
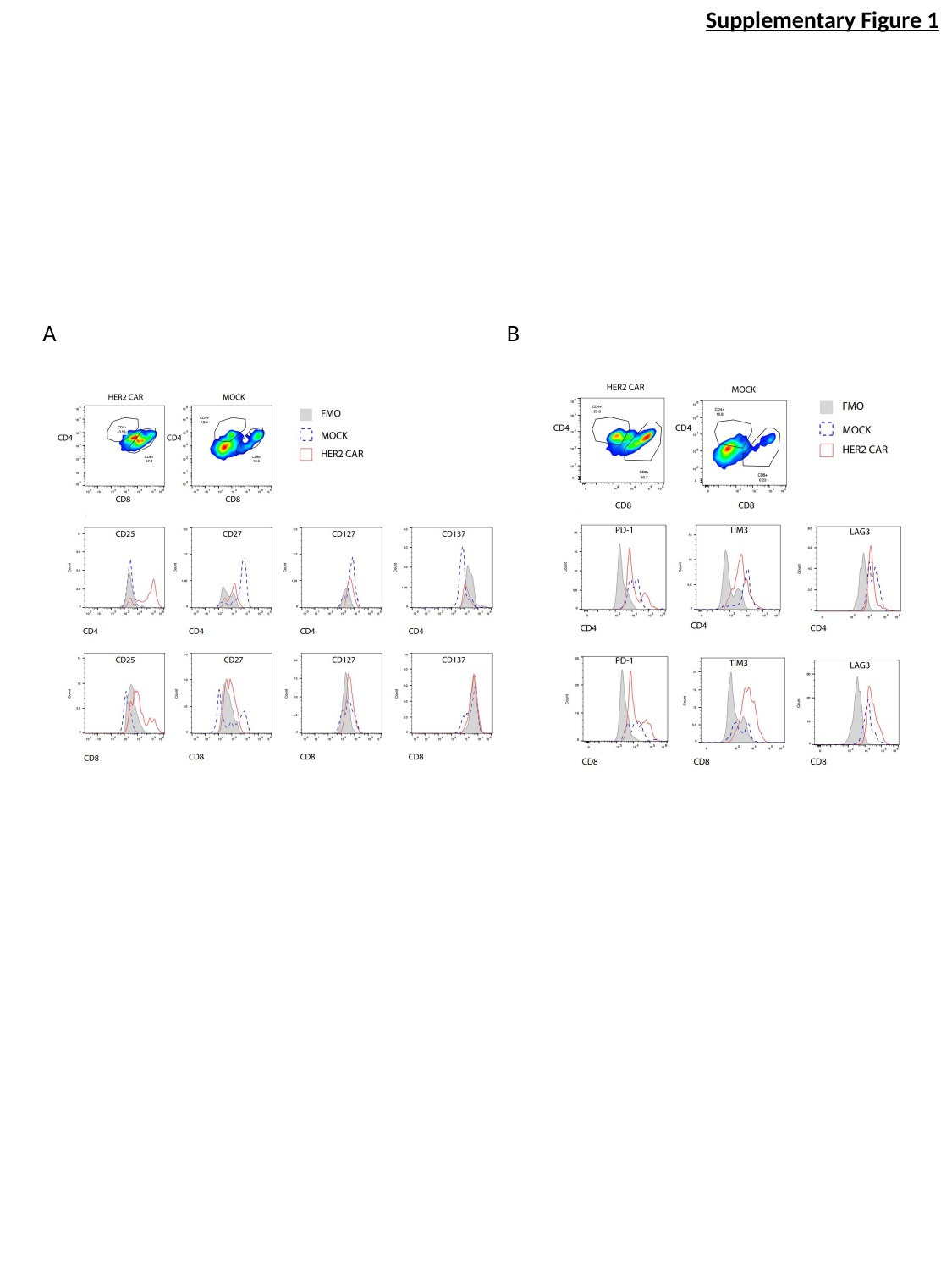

Supplementary Figure 1
A
B

Supplement: Supplementary file 1 — Figure S1. Characterization of HER2 CAR T cell phenotype. Human T cells were either mock transduced or transduced with HER2 CAR as previously described in Methods. Cells were taken from culture on Day 7 and stained for CD4, CD8, and either a panel of markers of T cell activation (A) or a panel of markers of T cell exhaustion (B). CAR+ T cells were identified by Protein-L staining as previously shown in Fig. 1. Histograms for each population of T cells are shown above, as well as the relevant FMO controls. (PPTX 389 kb) [file 40425_2018_340_MOESM1_ESM.pptx]

## Slide 1
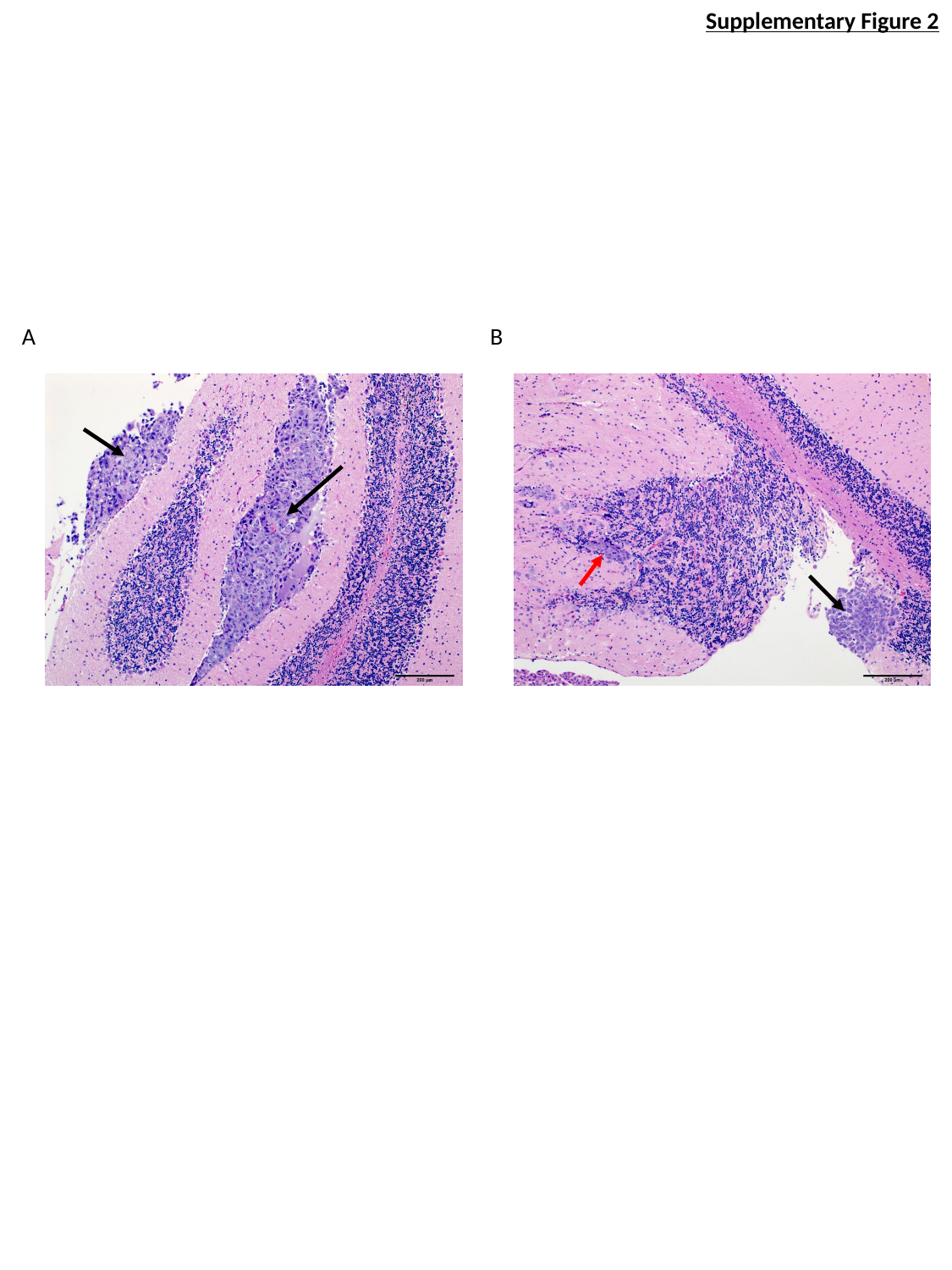

Supplementary Figure 2
A
B

Supplement: Supplementary file 2 — Figure S2. Medulloblastoma xenograft histology. NOD.Cg-Prkdcscid Il2rgtm1Wjl/SzJ (NSG) mice were injected with DAOY-GL tumor cells, as described in Methods, and treated with human CD19 CAR T cells intratumorally (A) or intravenously (B). Mice were euthanized at day 22 post-treatment and brain tissue was collected for histology. Brains were sectioned and stained using H&E. Images were taken using a digital slide scanner at 10X magnification, with representative results shown above. DAOY-GL cells mainly formed tumors along the periphery of the cerebellum (indicated by black arrows), but can also be seen infiltrating into the parenchyma adjacent to normal cerebellar cells (indicated by red arrow). (PPTX 5146 kb) [file 40425_2018_340_MOESM2_ESM.pptx]

## Slide 1
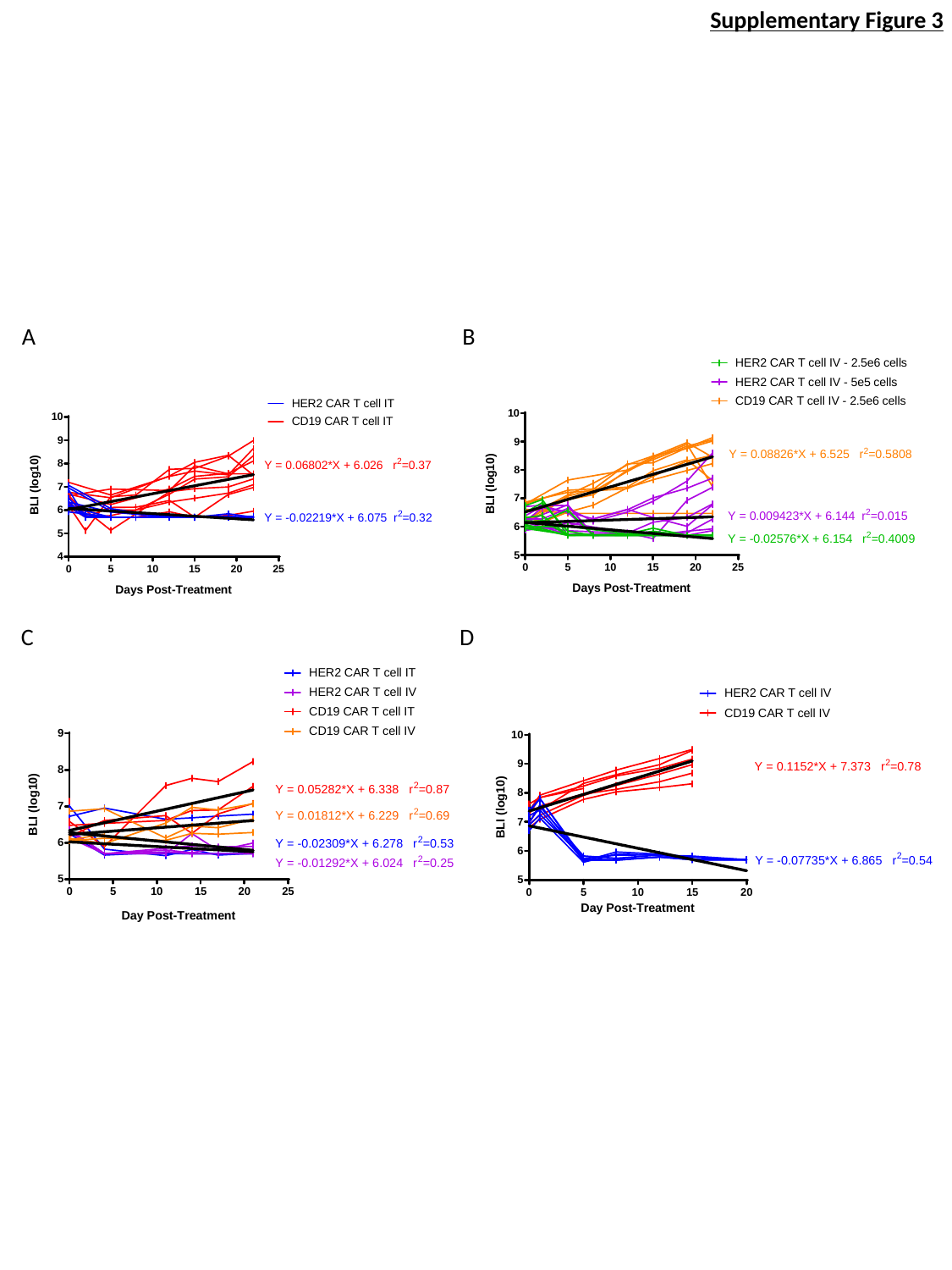

Supplementary Figure 3
A
B
C
D

Supplement: Supplementary file 3 — Figure S3. Linear regression data used for calculating statistics from Figs. 2, 3, 4, and 5. Data is presented as spider plots, with each line representing data from an individual mouse, and linear regression lines and equations overlaid. Fig. 2b. Fig. 3b. Fig. 4b. Fig. 5b. (PPTX 274 kb) [file 40425_2018_340_MOESM3_ESM.pptx]
